# Extraction Free RT-PCR Surveillance Testing and Reporting for SARS-CoV-2

**DOI:** 10.3390/covid3070075

**Published:** 2023-07-21

**Authors:** Patrick Carney, Tyler Duellman, Jia-Yi Chan, Lauren Wells, Michael Tessmer, Leah Frater-Rubsam, Molly Zeller, Mark Field, James Speers, Kelly Tyrrell, Luke Thompson, Michael Bondurant, Tami Morin, Tamra Dagnon, Brian Goff, Carissa Runde, Sandra Splinter-Bondurant, Charles Konsitzke, Patrick Kelly, Christopher Bradfield, Joshua Hyman

**Affiliations:** 1 Molecular and Environmental Toxicology, School of Medicine and Public Health, University of Wisconsin-Madison, Madison, Wisconsin 53706, USA; 2 Department of Oncology, School of Medicine and Public Health, University of Wisconsin-Madison, Madison, Wisconsin 53706, USA; 3 Biotechnology Center, University of Wisconsin-Madison, Madison, Wisconsin 53706, USA; 4 Division of Information Technology, University of Wisconsin-Madison, Madison, Wisconsin 53706, USA; 5 University Communications, University of Wisconsin-Madison, Madison, Wisconsin 53706, USA; 6 University Health Services, University of Wisconsin-Madison, Madison, Wisconsin 53706, USA

**Keywords:** SARS-CoV-2, COVID-19, RT-PCR, Surveillance

## Abstract

The COVID-19 pandemic necessitated sensitive, fast and inexpensive testing for the virus on university campuses across the nation in 2020 prior to the widespread availability of vaccines. Early testing efforts were limited by bottlenecks on reagents, low throughput testing options and slow return of test results. In this paper we detail the testing pipeline we established at the University of Wisconsin-Madison for rapid, inexpensive and sensitive surveillance testing for SARS-CoV-2 and highlight the strengths of the platform that would allow it to be applied to other disease surveillance projects, SARS-CoV-2 variant testing or future pandemics.

## 1. Introduction

The emergence of SARS-CoV-2 in late 2019 leading to the COVID-19 pandemic shuttered research laboratories across the country and necessitated innovation in viral testing that was sensitive, fast and inexpensive for clinical diagnostic and non-clinical surveillance applications. Further, testing needs and capabilities varied dramatically based on local resources. As a biotechnology core on a major public campus with over 45,000 students and staff our group was uniquely positioned to attempt to address local testing needs.

Initial diagnostic testing recommendations from the Centers for Disease Control and Prevention in early 2020 relied on the detection of two viral nucleocapsid targets (2019-nCoV_N1 and 2019-nCoV_N2) alongside a human positive control (RP) utilizing specific RNA extraction kits and a small number of RT-PCR Mastermix options[1]. The recommendations were also established for use in a 96-well format on Applied Biosystems 7500 Fast Dx Real-time PCR Instruments[1]. Given the paucity of diagnostics tests in early 2020 we sought to develop a sensitive, fast and inexpensive surveillance test for local use that avoided some of the common pitfalls of early testing. This included direct sample input to avoid competition with diagnostic labs sourcing RNA extraction reagents, PBS as a sample medium given the shortage of VTM, and multiplexing of RT-PCR targets on 384-well instruments to scale up testing capacity.

Numerous other groups have developed innovative extraction free testing platforms for SARS-CoV-2 detection and have repeatedly shown the process to be similarly sensitive to extraction based protocols[2]–[5]. Further, other groups have shown the equivalence of reverse-transcriptase loop mediated isothermal amplification (RT-LAMP) as compared to RT-PCR[6]–[8].

Here we detail the optimization of non-clinical extraction free RT-PCR testing for surveillance. Given the excellent clinical diagnostic testing options that became available in our area as well as early, widespread vaccination efforts our surveillance system was not fully utilized. The infrastructure in place would allow us to respond rapidly to an increased need for testing and can be adapted for other qPCR-based surveillance purposes such as respiratory illness screening, sexually transmitted infection screening, or response to a future potential pandemic agent.

## 2. Materials and Methods

### 2.1. Sample Collection and Inactivation

Unsupervised self-collection was performed using the Response Sample Kit (Genturi, Verona, WI). Instructions were printed and distributed in kit as shown (Figure 4B). Briefly, participants wash their hands, unscrew tube, swab inside of each nostril four times, break swab off in tube and replace cap on tube. The same is then placed back in the plastic bag. Bags containing samples are opened in BSC, tubes are checked for presence of swab and tight seal, then placed in autoclavable container with lid. Once filled and sealed the container is transferred to a 70 degree incubator for 30 minutes.

### 2.2. One-step RT-PCR

RT-PCR Mastermix is prepared using 3 uL 4x Taqpath 1-Step Multiplex Master Mix with Mustang Purple (Applied Biosystems), 1 uL N1-FAM Primer-Probe, 1 uL N2-ABY Primer-Probe, 1 uL RP-VIC Primer-Probe, and 3.5 uL Nuclease Free Water. 9.5 uL of this Mastermix is added to 3 uL Sample for a total reaction volume of 12.5 uL in one well of a 384-well plate. Primer-Probe sequences and concentrations can be found in Table 1 and were designed through Thermo Fisher Custom Oligos (Thermo Fisher, Waltham, MA). Cycling was performed on a QuantStudio 7 Pro Real-Time PCR 384-well Instrument (Thermo Fisher, Waltham, MA).

### 2.3. Data Analysis

Data was exported into the Design and Analysis Software Version 2.5 for the QuantStudio 6/7 Pro systems (Thermo Fisher, Waltham, MA). Amplification curve phenotypes were assessed and ΔRn thresholds were set at 1 for N1 and N2 and at 0.3 for RP. Cq values were exported and analyzed along with “Referral” and “No Action Needed” assessments as shown in Table 2.

## 3. Results

### 3.1. Development of a Multiplex SARS-CoV-2 qPCR Assay

**Figure 1.**
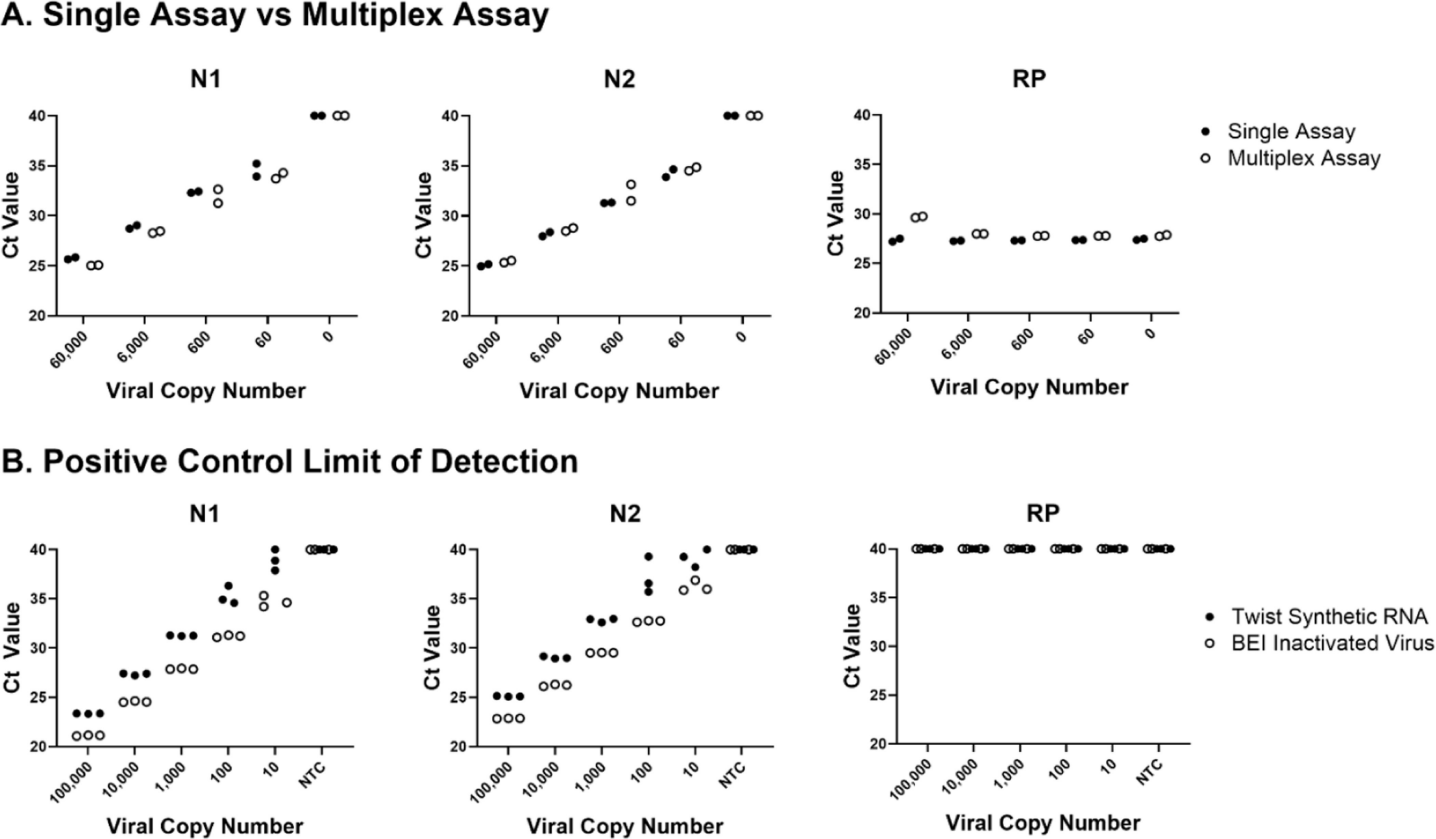
Development of Multiplex SARS-CoV-2 Assay. Our SARS-CoV-2 multiplex assay was developed based on the primer/probe set recommended by the CDC for ease of obtaining Emergency Use Authorization if needed. A. The primer/probe sets utilized show similar sensitivity for viral nucleocapsid targets (N1 and N2) and human control target (RP) over a dilution series of positive control SARS-CoV-2 nucleocapsid plasmid with a background of 30,000 copies/μL RP plasmid. B. Repeat testing of the multiplex assay with more physiologically relevant positive controls including synthetic RNA (Twist Biosciences) and heat-inactivated virus (ATCC) shows sensitive detection of viral genetic material over a dilution series. Of note, we observed Twist synthetic RNA degrades faster in solution than inactive viral samples, which may account for the slightly higher Ct values shown.

We developed our own qRT-PCR assay based on CDC recommended viral targets and controls[1]. To accomplish this, we designed a multiplex qRT-PCR assay for the viral targets (N1 and N2) and human control (RP) using Thermo Fisher custom assays (Thermo Fisher, Waltham, MA) as detailed in Table 1. Initial testing of these reagents against the CDC recommended 2019-nCoV_N_Positive Control plasmid (IDT, Coralville, IA) demonstrated similar performance using the probes alone or in combination as a multiplex assay (Figure 1A). Mock samples were prepared using the nCoV_N positive control plasmid at the indicated dilutions (copies/reaction) with the Hs_RPP30 control plasmid (IDT, Coralville, IA) spiked in at 40,000 copies/reaction to ensure the RP human control signal would not diminish the viral target signals. The multiplex assay performed similarly to single assays across a dilution series of positive controls and in the presence of a high amount of human control background. Further, we validated the multiplex assay against other positive controls including Twist Synthetic RNA (Control 2, 102024, Twist Bioscience, San Francisco, CA) and BEI Inactivated Virus (NR-52286 Heat Inactivated 2019-nCoV/USA-WA1/2020, ATCC, Manassas, VA) (Figure 1B). The assay shows similar sensitivity tested against both synthetic RNA and heat inactivated virus. It should be noted that dilutions were calculated based on the initial concentration of the product as it arrived which may account for the variability in Ct between the positive controls. In addition, we observed that Twist Synthetic RNA was more likely to degrade quickly with repeated freeze-thaws than the plasmid or heat-inactivated controls.

### 3.2. Testing of Direct RT-PCR from a Nasal Swab in PBS Medium

**Figure 2.**
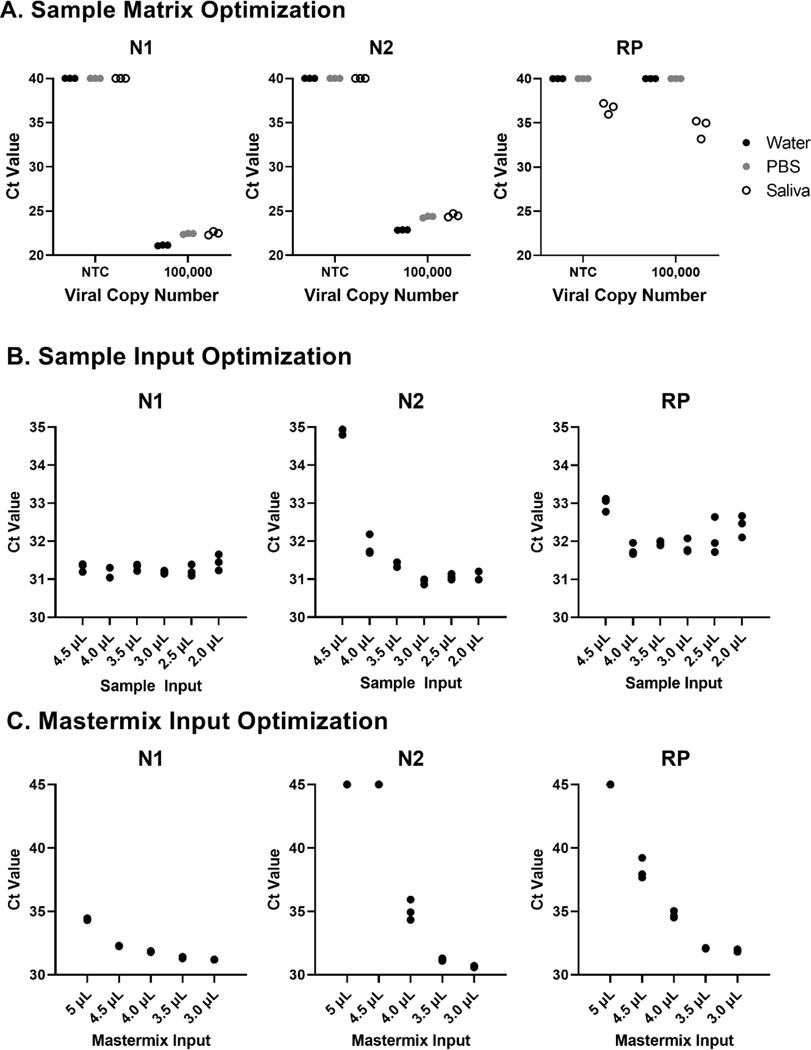
Matrix Testing and Optimization of Mastermix and Sample Volume A. Optimization of the multiplex SARS-CoV-2 involved testing assay performance in water, PBS and saliva showed similar sensitivity in all three solutions at 100,000 copies of heat-inactivated SARS-CoV-2 virus (ATCC). Based on this data we proceeded with a PBS-based nasal swab test as it maintained similar sensitivity to sample in water and was easier and safer for us to collect, inactivate and test. **B.** After selecting a PBS-based assay we optimized the amount of sample added to the qRT-PCR reaction. The N1 primer/probe set performed well across all conditions but there were some sensitivity issues with the N2 primer/probe set with high sample input. We decided to stick with 3 μL sample input; this gave us the lowest average Ct values across primer/probe sets and would allow for plenty of residual sample for repeat testing, variant testing and sequencing. **C.** We also optimized the amount of Taqpath Master Mix added to the reaction and determined 3 μL of Taqpath produced optimal Ct values across primer/probe sets.

Given the shortage of clinical testing materials (especially RNA extraction kits and VTM) and our desire to create a rapid, inexpensive surveillance test we developed a testing platform utilizing nasal swabs in a PBS medium. Initial testing with BEI inactivated virus showed similar sensitivity of the assay using water, PBS and saliva as a medium (Figure 2A). We elected to proceed with nasal swab testing in a PBS medium based on the ability to source a large number of pre-packaged PBS-filled tubes and nasal swabs (described in more detail later). Following optimization testing, we arrived at 3 uL of PBS-based sample input and 3 uL of Taqpath Mastermix (Thermo Fisher, Waltham, MA) as optimal volumes for our direct qRT-PCR testing (Figure 2B-C).

### 3.3. Test Kits and Testing Pipeline

**Figure 3.**
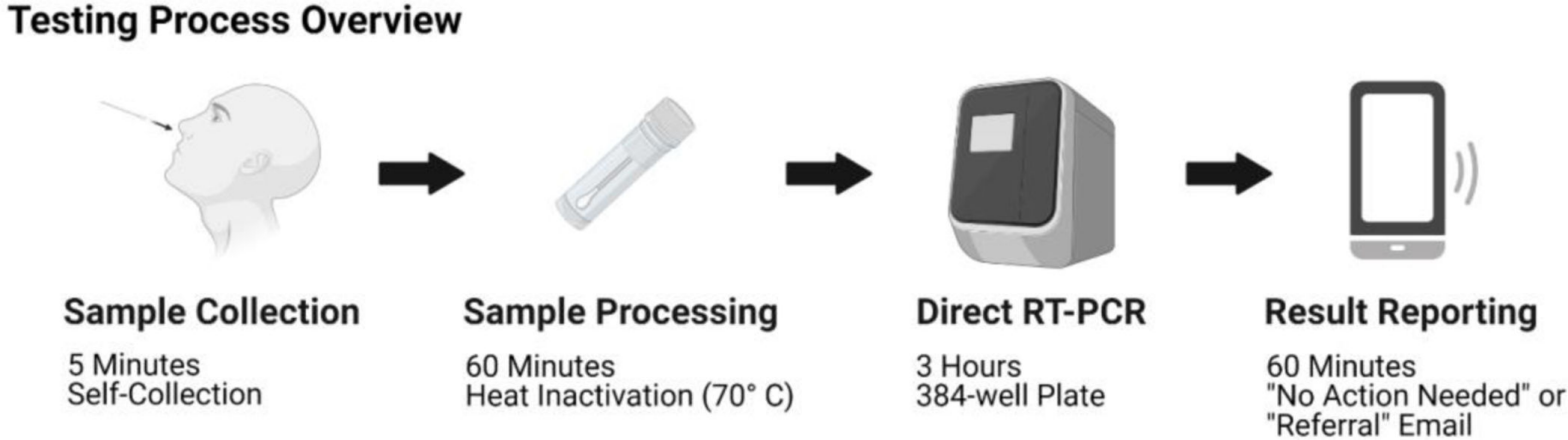
Testing Process. This schematic depicts the extraction free surveillance testing workflow we developed. Briefly, the participant registers the testing kit to their campus ID using the QR code provided and their mobile device, self-collects a nasal specimen, places it in a screw-cap tube containing 1 mL of PBS and submits the test. The samples are handled in a biological safety cabinet and heat-inactivated at 70° C for 30 minutes. 3 μL sample is then added directly to the qRT-PCR mastermix in a 384-well plate which is run on a Quantstudio 7 Pro. Data analysis is performed and a .csv file is uploaded with sample ID and an interpretation of “No Action Needed” for negative tests or “Referral” for positive tests or tests where the internal control fails. The test results are then matched to the campus ID and email of the participant who registered the test and the result sent by email. The entire process is intended to be completed in 6–8 hours and capacity can be easily increased with the use of robotics for sample handling. Created with BioRender.com.

**Figure 4.**
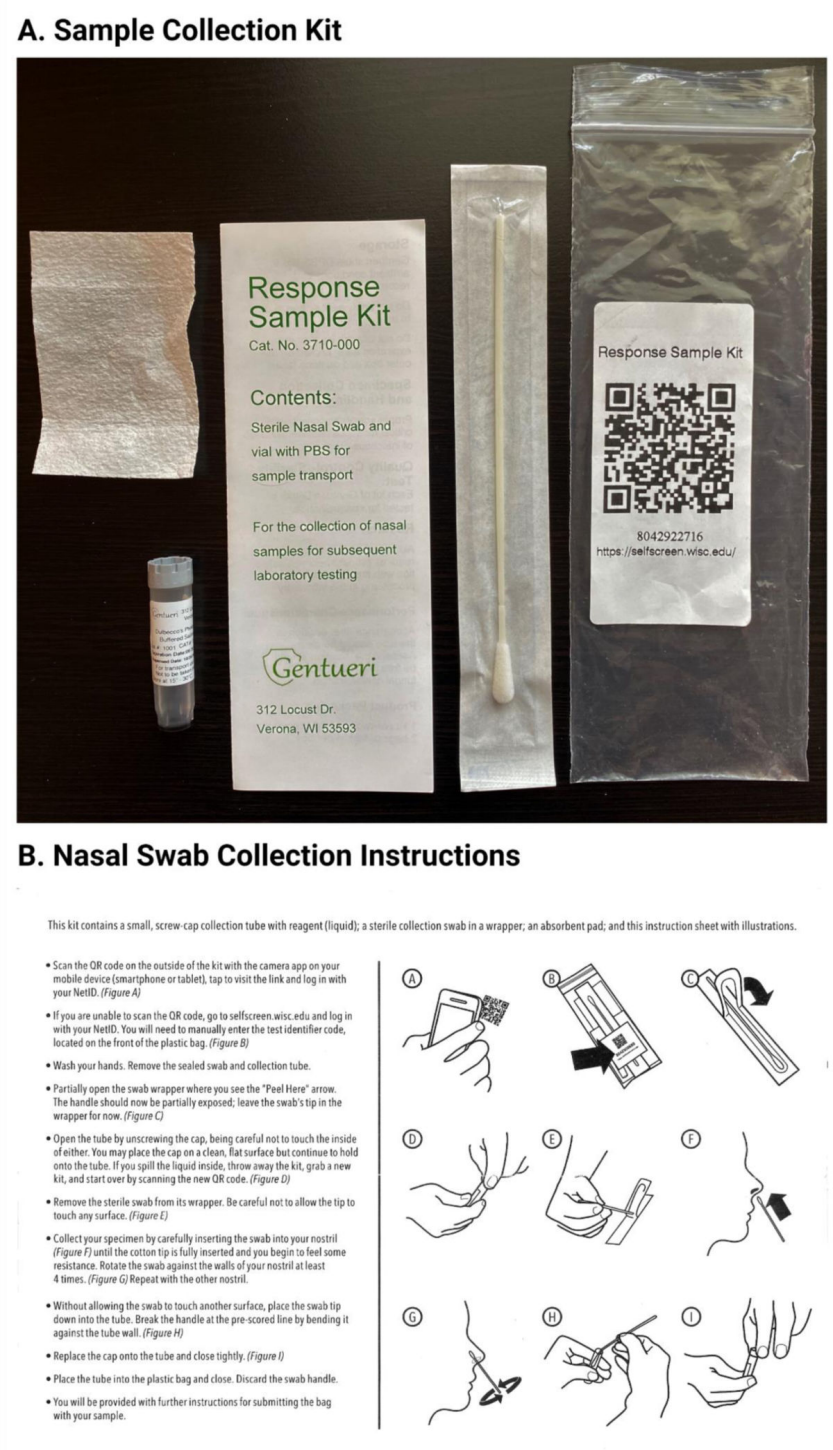
Sample Collection Kit A. The sample collection kit is pictured. The entire kit is delivered in a plastic bag with a QR code and unique test ID which allows the user to register the kit by going to https://selfscreen.wisc.edu. The kit consists of a flocked nasal swab, a 1.5 mL screw-cap tube pre-filled with 1 mL of sterile PBS, a test kit content insert and an absorbent pad in the event of sample spilling. B. The nasal swab collection instructions contain clear imagery and instructions to show the participant exactly how to register the kit and self-collect their nasal specimen. The instructions are also available in other languages. Created with BioRender.com

An overview of the testing process is pictured in Figure 3. Testing kits were purchased locally (Gentueri, Verona, WI) and included a flocked nasal swab in a protective pouch, a barcoded sample tube pre-filled with 1.5 mL PBS, an absorbent pad, a safety insert and sampling instructions all in a plastic bag with a QR code on the front (Figure 4A). One limitation of early SARS-CoV-2 testing was the requirement for supervised collection to perform diagnostic clinical testing; this test was designed to be self-collected to reduce the need for staffing a collection center and ease of access for participants. Self-collection steps are detailed in Figure 4B.

**Figure 5.**
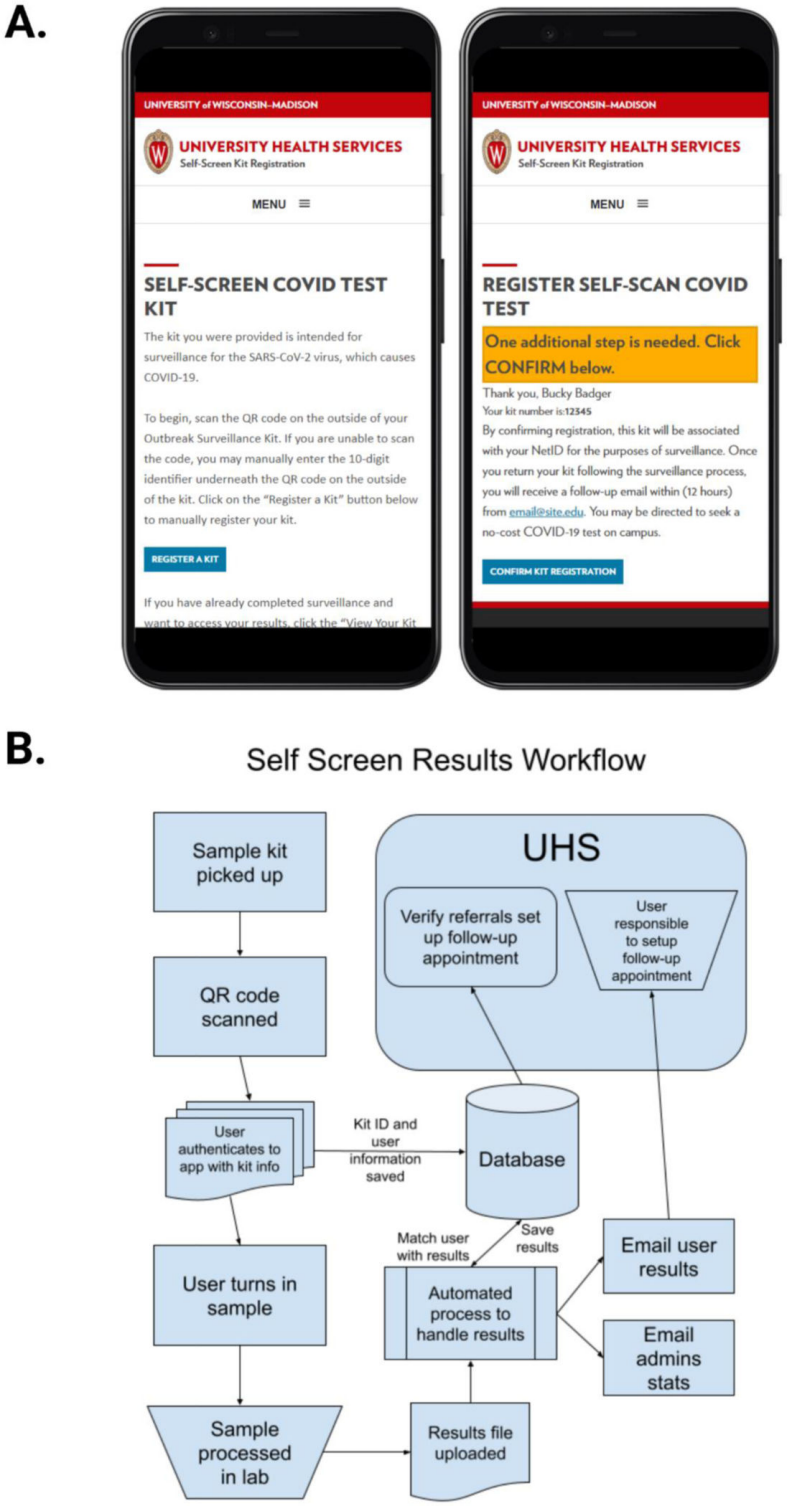
Testing Kit Registration and Testing Result Workflow A. A mockup of the self-screen registration page is shown on a mobile device with a welcome screen to register the kit and a confirmation screen to link the scanned kit to the user’s ID and preliminary instructions on what follow up may be required. B. The self-screen workflow is depicted showing how samples are linked to user identifiers. University Health Services (UHS) is notified of the results to verify individuals needing follow up and emails are sent to the user with instructions that indicate if follow up is necessary. Created with BioRender.com.

First, participants are instructed to scan the QR code on the bag with their smartphone which opens a website designed by the UW-Madison Division of Information Technology (DoIT) (Figure 5A). This provides a mechanism for UW-Madison staff and students to automatically link the kit barcode to their campus ID within the University Health Services record system and notifies UHS that the participant is submitting a sample. Participants are also able to use a computer to manually enter a kit ID if they didn’t have a smart phone or device with a working camera. This process increases the chance of user error by entering the wrong kit number, but steps were taken later during the automated process to try to validate any errors. Participants remove the swab from its protective pouch, swab the inside of each nostril 4 times and break the flocked swab off into the PBS-filled tube. Finally each participant seals the tube, places the sample in the specimen bag and submits it. Self-collection takes roughly five minutes. Samples are placed in a collection box to be transported to the processing facility that day. Once samples arrive at the processing facility, they are removed from the specimen bag in a biological safety cabinet and placed in an incubator to be heat inactivated at 65C for half an hour before being transferred directly to a 384-well plate and combined with the multiplex primer/probe assay and Taqpath Mastermix for qRT-PCR. Following qRT-PCR the data is analyzed by a member of the testing facility and a .csv file is uploaded with results indicating “Referral” for samples in which viral targets were positive or “No Action Needed” for samples in which there was no viral target. This terminology was selected specifically in keeping with this test being utilized for widespread non-diagnostic surveillance testing such that the university could screen a large number of people rapidly and recommend follow-up diagnostic testing for a much smaller subset.

The upload location is monitored by an automated process watching for new .csv files. When a new file is found it is parsed and the test results are merged with the matching user. Known controls (guaranteed “referral” or “non-referral”) in the results are validated and administrators are alerted when a result didn’t match expected outcomes (Figure 5B). The automated process then sends out a predefined email message alerting users of their results and notifies University Health Services of basic statistics from that run (e.g. total samples, total referrals, total non-referrals, missing kits, etc.). Users with a referral result are instructed to create a follow-up appointment with UHS and are able to return to the web application to get more information on next steps.

## 4. Discussion

The SARS-CoV-2 pandemic challenged the scientific community to respond and innovate to meet the need for viral testing. Clinical diagnostic testing in labs with CAP/CLIA approval was the early gold standard in testing but its reliance on RNA extraction reagents, approved nasal swabs and transport medium and the need for well-trained personnel to staff the testing labs meant that availability of these tests early on was scarce. We sought to develop a non-clinical surveillance testing pipeline that bypassed these restrictions for rapid, inexpensive and sensitive testing results to inform local decision-making. Our results show that our extraction free multiplex RT-PCR testing platform retains sensitivity while bypassing RNA extraction, providing a testing option that is faster, cheaper and more scalable than traditional extraction-based clinical diagnostic testing.

Our group was specifically tasked with delivering a testing process that can be completed in 6–8 hours for rapid turnaround of surveillance test results to inform decision making on our campus. We accomplished this by partnering with campus healthcare workers (University Health Services) and campus information technology specialists (DoIT) to create our testing pipeline. One major strength of our innovation here is the use of self-collection nasal swabs for obtaining specimen, which reduces the need to staff and expose testing center personnel. Additionally, the unique QR code linked to a campus ID allows for participants to register their kit safely and securely such that only campus healthcare information technology staff can link the kit back to them and report their test results securely through email. By creating an extraction free direct RT-PCR testing process we estimate the testing facility could manually process about 1000 samples in one day with 6–8 hour turnaround on those samples using only two testing facility staff members working full time. This process could be scaled up dramatically with the use of robotics for RT-PCR plate preparation.

The result of this work is the establishment of infrastructure for rapid, sensitive and inexpensive swab-based testing. This testing platform could be used for ongoing SARS-CoV-2 surveillance, in response to another outbreak or the emergence of a concerning SARS-CoV-2 variant. In addition the platform could rapidly be repurposed for testing for another potential pandemic agent, seasonal illnesses such as influenza, or detection of other infections such as sexually transmitted infections.

## Figures and Tables

**Table 1.** SARS-CoV-2 Multiplex Assay Reagents List of the primer and probe names and sequences as used in the SARS-CoV-2 multiplex assay.

Primer/probe Name	Gene	Sequence (5′ to 3′)	Reporter Dye	Quencher	Final Concentration (nM)
2019-nCoV_N1-FWD	SARS-CoV-2 N	GACCCCAAAATCAGCGAAAT	ABY	QSY	500
2019-nCoV_N1-REV		TCTGGTTACTGCCAGTTGAATCTG			500
2019-nCoV_N1-ABY		ACCCCGCATTACGTTTGGTGGACC			250
2019-nCoV_N2-FWD	SARS-CoV-2 N	TTACAAACATTGGCCGCAAA	FAM	QSY	500
2019-nCoV_N2-REV		GCGCGACATTCCGAAGAA			500
2019-nCoV_N2-FAM		ACAATTTGCCCCCAGCGCTTCAG			250
RP-FWD	RPP30	AGATTTGGACCTGCGAGCG	VIC	QSY	100
RP-REV		GAGCGGCTGTCTCCACAAGT			100
RP-VIC		TTCTGACCTGAAGGCTCTGCGCG			50

**Table 2.** Presence/Absence Caller Settings. Presence/Absence settings used on in the Design and Analysis Presence/Absence module to make a call and deliver an assessment result.

Presence Targets	Absence Targets	Call	Assessment
N1, N2, RP		Presence	Referral
N1	N2, RP	Presence	Referral
N2	N1, RP	Presence	Referral
N1, RP	N2	Presence	Referral
N2, RP	N1	Presence	Referral
N1, N2	RP	Presence	Referral
	N1, N2, RP	Inconclusive	Referral
RP	N1, N2	Absence	No Action Needed

## Data Availability

The data presented in this study are available on request from the corresponding author.
